# Sex-specific differences in KCC2 localisation and inhibitory synaptic transmission in the rat hippocampus

**DOI:** 10.1038/s41598-022-06769-5

**Published:** 2022-02-24

**Authors:** Daniele C. Wolf, Nathalie T. Sanon, Alexandra O. S. Cunha, Jia-Shu Chen, Tarek Shaker, Abdul-Rahman Elhassan, Antônia Sâmia Fernandes do Nascimento, Graziella Di Cristo, Alexander G. Weil

**Affiliations:** 1 Centre de Recherche, Centre Hospitalier Universitaire (CHU) Sainte-Justine, Département de Pédiatrie, Université de Montréal, 3175 Chem. de La Côte-Sainte-Catherine, Montreal, QC H3T 1C5 Canada; 2 Département de Neurosciences, Université de Montréal, 2900, Boul. Édouard-Montpetit, Montreal, QC H3T 1J4 Canada; 3 Department of Physiology, School of Medicine of Ribeirão Preto, University of Sao Paulo, Campus da Usp, Ribeirao Preto, SP 14040-900 Brazil; 4 The Warren Alpert Medical School of Brown University, 222 Richmond St, Providence, RI 02903 USA; 5 Department of Physiology, Neuroscience and Mental Health Institute, University of Alberta, 2-132 Li Ka Shing Centre for Health Research Innovation, Edmonton, AB T6G 2E1 Canada; 6 Neurosurgery Service, Department of Surgery, Université de Montréal, 3175 Chem. de la Côte-Sainte-Catherine, Montreal, QC H3T 1C5 Canada

**Keywords:** Cellular neuroscience, Development of the nervous system, Neural circuits, Neuronal physiology

## Abstract

Sexual differentiation of the brain is influenced by testosterone and its metabolites during the perinatal period, when many aspects of brain development, including the maturation of GABAergic transmission, occur. Whether and how testosterone signaling during the perinatal period affects GABAergic transmission is unclear. Here, we analyzed GABAergic circuit functional markers in male, female, testosterone-treated female, and testosterone-insensitive male rats after the first postnatal week and in young adults. In the hippocampus, mRNA levels of proteins associated with GABA signaling were not significantly affected at postnatal day (P) 7 or P40. Conversely, membrane protein levels of KCC2, which are critical for determining inhibition strength, were significantly higher in females compared to males and testosterone-treated females at P7. Further, female and testosterone-insensitive male rats at P7 showed higher levels of the neurotrophin BDNF, which is a powerful regulator of neuronal function, including GABAergic transmission. Finally, spontaneous GABAergic currents in hippocampal CA1 pyramidal cells were more frequent in females and testosterone-insensitive males at P40. Overall, these results show that perinatal testosterone levels modulate GABAergic circuit function, suggesting a critical role of perinatal sex hormones in regulating network excitability in the adult hippocampus.

## Introduction

Female animal models have been consistently excluded from most basic neuroscience studies for decades^1–4^ despite the well documented sex bias observed in many neurological and neuropsychiatric conditions, such as autism spectrum disorders (ASD), epilepsy, mood disorders, multiple sclerosis, Alzheimer’s and Parkinson’s disease^5–9^. Therefore, considering sex as a biological variable (SABV) in the design and analysis of basic and clinical research is extremely important when making treatment decisions for both men and women^3,10,11^.

Hormonally influenced sex differences are caused by either activational or organizational effects of hormones^12–16^. Briefly, circulating gonadal hormones can have acute and transitory effects throughout life (activational effects), whereas exposure to gonadal hormones during developmental phases might result in persistent sex differences (organizational effects)^17^. Many molecular and cellular processes are influenced by gonadal hormones in the developing brain, including gene expression, cell birth and death, neurite outgrowth, synaptogenesis, and synaptic activity^18^. Extensive clinical observations suggest that males have a two-to-four times higher risk of being affected by neurodevelopmental disorders than females^19^. It has been suggested that gonadal hormones lead to functional difference in neuronal circuit development, thus contributing to sex bias in neurodevelopmental disorders^5,20^.

The balance between excitatory and inhibitory circuits is fundamental for all aspects of brain function^21^. Gamma-Aminobutyric acid (GABA), the principal inhibitory neurotransmitter, plays an important role in maintaining the inhibitory tone that counterbalances neuronal excitation. The inhibitory action of GABA relies on the inflow of chloride ions (Cl^−^), which hyperpolarises neurons in the brain. However, in early development, GABA signaling induces outward Cl^−^ currents, resulting in membrane depolarization. The postnatal shift in GABA function relies on the developmentally regulated expression of the Na^+^–K^+^–2Cl^−^ cotransporter 1 (NKCC1) and the K^+^–Cl^−^ cotransporter 2 (KCC2). Immature neurons accumulate Cl^−^ due to a high level of NKCC1, a Cl^−^ importer, resulting in Cl^−^ efflux and membrane depolarization upon GABA_A_Rs opening. Conversely, mature neurons express higher levels of the Cl^−^ exporter, KCC2, which results in lower intracellular Cl^−^ concentration which drives Cl^−^ influx through GABA_A_Rs and leads to hyperpolarization^22,23^. Interestingly, recent studies showed that the timing of KCC2 and NKCC1 expression, and consequently the onset of mature inhibitory GABAergic neurotransmission during brain development, is sex dependent and occurs earlier in females compared to males^24–27^. However, it is unknown whether the observed sex difference in chloride transporter expression is dependent on perinatal hormones.

In this study, we explored the role of perinatal testosterone on GABAergic inhibition, by analyzing the expression of different molecular determinants of GABAergic neurotransmission and recording spontaneous and miniature GABAergic currents in hippocampal CA1 pyramidal neurons in males, females, masculinized females, and testosterone-insensitive males. Exogenous testosterone administration during sensitive periods of brain development induces adult male sexual behavior and brain anatomical patterns in female rodents^28,29^. On the other hand, the testicular feminization mutation (Tfm) is a naturally occurring point mutation of the gene encoding the androgen receptor (AR) that renders the Tfm male rat insensitive to physiological levels of androgens^30^. This latter approach allowed us to explore the organizational sex differences under baseline conditions, which are often undetected.

## Results

### Profile of neonatal testosterone and estradiol at birth

**Figure 1:**
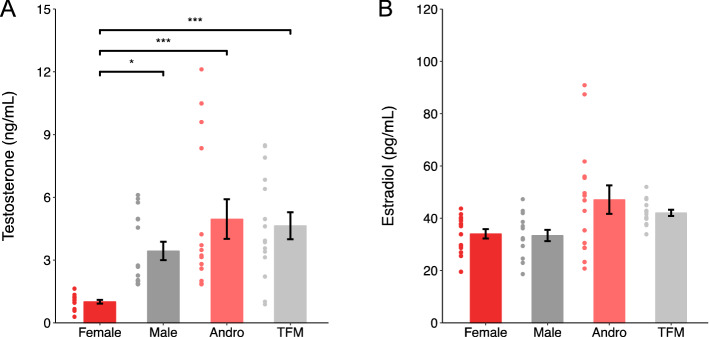
Testosterone is significantly lower in females whereas estradiol levels are similar between sex groups. (**A**) Bar graph shows higher testosterone levels in all sexes, except for female rat pups (F: 1.01 ± 0.09 ng/mL, n = 15 rats from 3 litters; M: 3.44 ± 0.44 ng/mL, n = 15 rats from 3 litters; A: 4.96 ± 0.95 ng/ml, n = 14 rats from 4 litters; TFM: 4.64 ± 0.65 ng/Ml, n = 15 rats from 5 litters; One-way ANOVA with Tukey’s post hoc analysis, F × M: p = 0.028; F × A: p < 0.001; F × TFM: p < 0.001). (**B**) Bar graph shows no significant differences in levels of estradiol between all groups (F: 34.0 ± 1.81 pg/mL, n = 15 rats from 3 litters; M: 33.4 ± 2.14 pg/mL, n = 15 rats from 3 litters; A: 47.1 ± 5.47 pg/mL, n = 14 rats from 4 litters; TFM: 42.1 ± 1.18 pg/mL, n = 15 rats from 5 litters; One-way ANOVA with Tukey’s post hoc analysis, F × M: p = 0.995; F × A: p = 0.080; F × TFM: p = 0.140). Dots represent individual data points. *M* males, *F* females, *A* andro/testosterone-treated females, *TFM* testosterone-insensitive males. Graphs represent mean ± SEM.

In rats, testicular testosterone production peaks in the late gestational period (E18) and is high throughout the first few postnatal days^18^. Therefore, to characterize the levels of plasma testosterone and estradiol in male, female, testosterone-treated female, and testosterone-insensitive male rats, samples were taken from spontaneously delivered pups within 24 h after birth, i.e., P0. We found that plasma testosterone levels were elevated in male, testosterone-treated female, as well as in testosterone-insensitive male rat pups when compared to female rat pups at birth (Fig. 1A; One-way ANOVA with Tukey’s post hoc analysis, F × M: p = 0.028; F × A: p < 0.001; F × TFM: p < 0.001). Conversely, there were no differences in plasma estradiol levels across the groups (Fig. 1B; One-way ANOVA with Tukey’s post hoc analysis, F × M: p = 0.995; F × A: p = 0.080; F × TFM: p = 0.140).

### Perinatal testosterone determined sexual developmental markers

**Figure 2:**
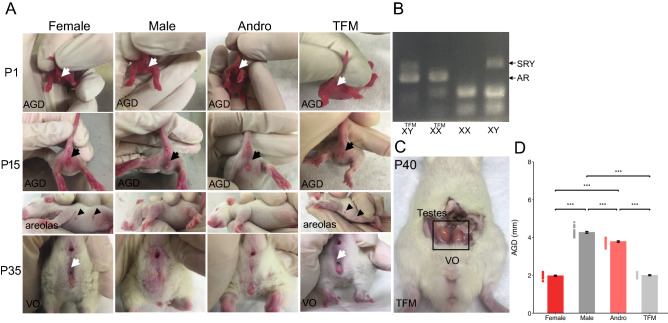
Testosterone levels affect sexual developmental markers. (**A**) Photographs show representative anogenital distances AGD (white arrows) observed in male, female, testosterone-treated females, and testosterone-insensitive male rats at P1, 15 and 35. Presence or absence of areolas are represented by black arrowheads. Vaginal opening (VO) is observed (white arrows) at P35. (**B**) PCR confirming the genotype of each animal (Tfm vs. wt alleles for androgen receptor (AR). (**C**) Presence of testes in testosterone-insensitive males despite the feminine external phenotype. (**D**) Bar graph shows significant differences in AGD length between sex groups (F: 1.98 ± 0.03 mm, n = 24 rats from 5 litters; M: 4.27 ± 0.05 mm, n = 25 rats from 5 litters; A: 3.79 ± 0.04 mm, n = 23 rats from 7 litters; TFM: 2.00 ± 0.03 mm, n = 17 rats from 6 litters; One-way ANOVA with Tukey’s post hoc analysis, F × M, F × A TFM × M, TFM × A: p < 0.0001). Dots represent individual data points. *F* females, *M* males, *A* andro/testosterone-treated females, *TFM* testosterone-insensitive males. Graphs represent mean ± SEM.

Sexual developmental marker analysis revealed that testosterone affected the anogenital distance (AGD) length and secondary sexual characteristics from birth until puberty (Fig. 2). At P1, we found smaller AGDs in females and testosterone-insensitive males, and larger AGDs in males and testosterone-treated females (Fig. 2A,D; One-way ANOVA with Tukey’s post hoc analysis, F × M, F × A TFM × M, TFM × A: p < 0.0001). At P15, we found that areolas were either absent or normal (prominent). All females and testosterone-insensitive males displayed normal areolas. Conversely, areolas were not detected in any male or testosterone-treated female offspring (Fig. 2A). At P35, sexual maturity was assessed in female and testosterone-treated female rats by inspecting the vaginal opening (VO). Testosterone treatment during the perinatal period affected the VO. Dissection of testosterone-insensitive male offspring at P40 revealed testes, confirming their male genotype (Fig. 2B,C). Overall, these results showed that the presence of androgens at the time of birth, like it occurs in normal males or in females treated perinatally with exogenous testosterone, resulted in the development of male-like phenotypes, which include development of secondary sexual characteristics. These results are in accordance with previous data showing that, while the Tfm mutation does not render AR completely non-functional, the decrease in function by 85–90%^30^ is enough to generate an entirely feminine external phenotype^31^. Notably, gonadal steroid production is intact in the Tfm male, resulting in testosterone levels that are rather high yet within the normal range^32^ as show in Fig. 1A.

### Perinatal testosterone signaling did not affect mRNA levels of major molecular determinants of GABAergic neurotransmission

**Figure 3:**
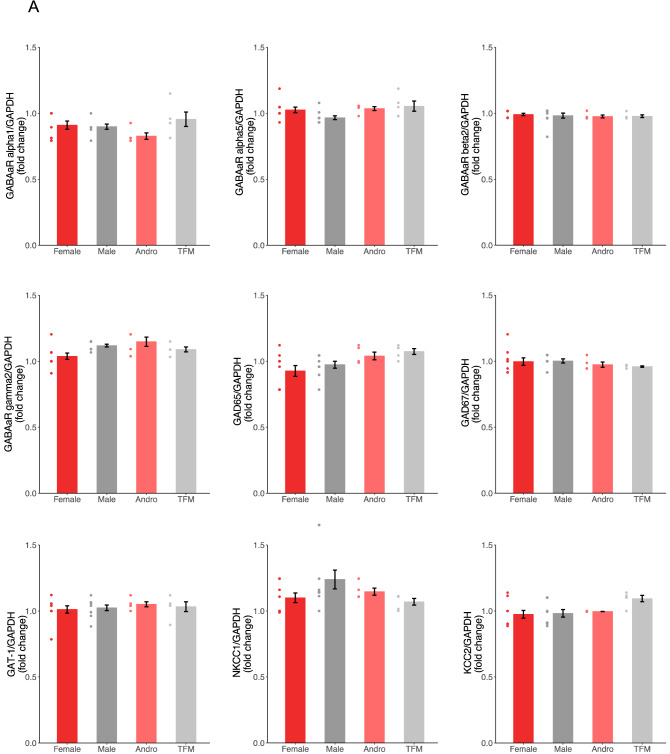

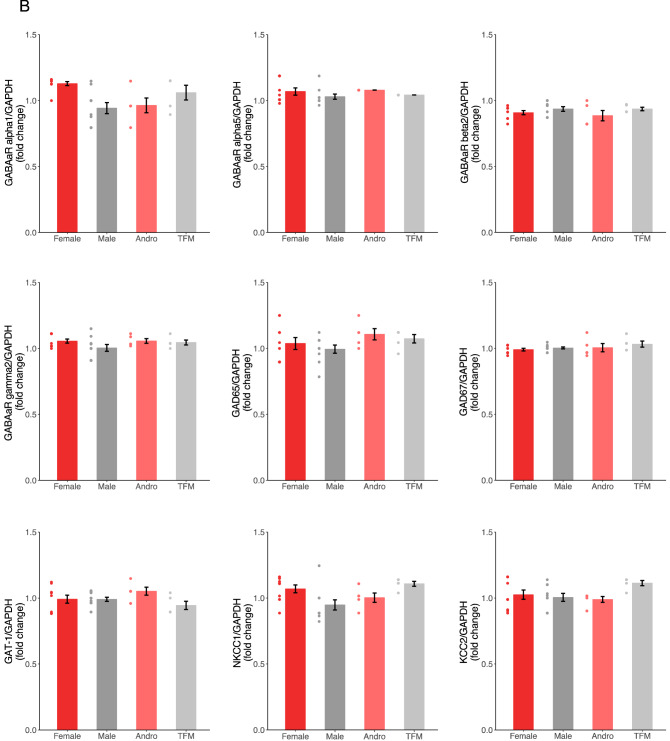
Testosterone does not affect mRNA levels of major GABAergic neurotransmission determinants. (**A**,**B**) Fold-change values in mRNA expression of GABA signaling components extracted from rat hippocampus at P7 (**A**) and P40 (**B**) do not show significant differences between groups (F: n = 10 rats from 3 litters; M: n = 10 rats from 3 litters; A: n = 5 rats from 3 litters; TFM: n = 5 rats from 3 litters; One-way ANOVA with Tukey’s post hoc analysis, p > 0.05, fold change > 2 or < 0.5 for all comparisons at P7; One-way ANOVA with Tukey’s post hoc analysis, p > 0.05, fold change > 2 or < 0.5 for all comparisons at P40). Dots represent individual data points. *F* females, *M* males, *A* andro/testosterone-treated females, *TFM* testosterone-insensitive males. Graphs represent mean ± SEM.

Previous studies have shown fluctuations in expression levels of GABA signaling components between sexes^33,34^. Therefore, to characterize the GABA signaling components in our four experimental groups, we quantified by RT-qPCR the mRNA expression levels of GABA-A receptor subunits (α1, α5, β2, γ2), GABA transporter (GAT-1), GABA synthesis (GAD65 and GAD67) and Chloride co-transporters (NKCC1 and KCC2) in hippocampal samples collected from the four groups at P7, when GABAergic circuits are still developing, and P40, when GABAergic transmission is considered mature. We found no significant difference in mRNAs expression levels for any GABA signaling components in all sex groups at both ages (Fig. 3A,B; One-way ANOVA with Tukey’s post hoc analysis, p > 0.05, fold change > 2 or < 0.5 for all comparisons). Therefore, testosterone signaling at birth seemed to not have any effects on the transcription of major molecular determinants of GABAergic signaling.

### Testosterone signaling delayed membrane localisation of KCC2 during the first postnatal week

**Figure 4:**
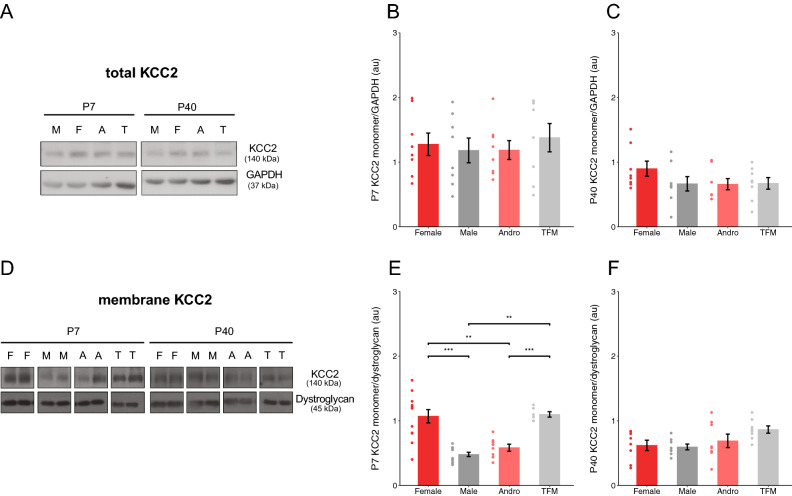
Perinatal testosterone limits KCC2 localisation at the membrane. (**A**) Western blot membrane of total KCC2 (images were cropped from the same gel). (**B**,**C**) Quantification of total KCC2 expression levels (monomer band at 140 kDa) in the hippocampus of different experimental groups do not show significant differences at P7 (**B**) (F: 1.28 ± 0.17 au, n = 8 rats from 3 litters; M: 1.18 ± 0.19 au, n = 8 rats from 3 litters; A: 1.19 ± 0.15 au, n = 8 rats from 3 litters; TFM: 1.38 ± 0.22 au, n = 8 rats from 3 litters; One-way ANOVA with Tukey’s post hoc analysis, p = 0.926) or at P40 (**C**) (F: 0.90 ± 0.12 au, n = 8 rats from 3 litters; M: 0.67 ± 0.11 au, n = 8 rats from 3 litters; A: 0.66 ± 0.09 au, n = 8 rats from 3 litters; TFM: 0.67 ± 0.09 au, n = 8 rats from 3 litters; One-way ANOVA with Tukey’s post hoc analysis, , p = 0.355). (**D**) Western blot membrane of KCC2 in membrane fractions (images were cropped from different gels). (**E**,**F**) Quantification of KCC2 monomer expression levels in the membrane fractions show sex differences in the hippocampus at P7 (E) (F: 1.07 ± 0.10 au, n = 12 rats from 4 litters; M: 0.48 ± 0.03 au, n = 12 rats from 5 litters; A: 0.58 ± 0.05 au, n = 8 rats from 3 litters; TFM: 1.10 ± 0.04 au, n = 5 rats from 3 litters; one-way ANOVA with Tukey’s post hoc analysis, F × M: *p* < 0.001; F × A: *p* = 0.001, TFM × M: *p* < 0.001; TFM × A: *p* = 0.001). Conversely, no sex differences were found at P40 (F) (F: 0.62 ± 0.08 au, n = 8 rats from 3 litters; M: 0.60 ± 0.04 au, n = 8 rats from 4 litters, A: 0.69 ± 0.11 au, n = 8 rats from 3 litters; TFM: 0.87 ± 0.06 au, n = 8 rats from 3 litters; One-way ANOVA with Tukey’s post hoc analysis, p = 0.121). Each lane represents a different animal. Blot shows representative samples of different sex groups. Dots represent individual data points. *F* females, *M* males, *A* andro/testosterone-treated females, *T* TFM/testosterone-insensitive males. Graphs represent mean ± SEM.

The increase of KCC2 expression during the first two postnatal weeks underlies the onset of powerful inhibitory neurotransmission in the hippocampus^21–23^. To evaluate the impact of testosterone on KCC2 expression, we quantified the monomeric (140KDa) form of KCC2 at two developmental ages, P7 and P40, by western blot. We did not observe any significant differences in KCC2 expression levels in whole cell lysates between any of the groups for KCC2 monomer band (Fig. 4A, Supplementary Fig. S1B,C; One-way ANOVA with Tukey’s post hoc analysis, p = 0.926 at P7; One-way ANOVA with Tukey’s post hoc analysis, p = 0.355, at P40) in contrast to what previously reported^24,25^. While KCC2 protein can be found both in cytoplasmic and plasmalemmal compartments, KCC2-dependent Cl-extrusion in neurons is mostly reliant on KCC2 localization at the membrane. Therefore, to investigate whether KCC2 localization at the membrane was sex dependent, we quantified KCC2 monomer levels in the membrane fractions from hippocampi of P7 and P40 rats (Fig. 4D, Supplementary Fig. S1). At P7, we found that KCC2 monomer levels in the membrane fractions were significantly higher in female when compared to males and testosterone-treated female rats (Fig. 4E; One-way ANOVA with Tukey’s post hoc analysis, F × M: *p* < 0.001; F × A: *p* = 0.001). Furthermore, testosterone-insensitive male rats showed higher KCC2 monomer levels in the membrane fractions when compared to male and testosterone-treated female rats at the same age (Fig. 4E; One-way ANOVA with Tukey’s post hoc analysis, TFM × M: *p* < 0.001; TFM × A: *p* = 0.001). Conversely, at P40 we observed no significant difference in KCC2 monomer levels in the membrane fractions between groups (Fig. 4F; One-way ANOVA with Tukey’s post hoc analysis, p = 0.121). Overall, our results showed that, during the first postnatal week, higher testosterone signaling was associated with lower KCC2 membrane localization.

### BDNF expression was higher in females and testosterone-insensitive males during the first postnatal week

**Figure 5:**
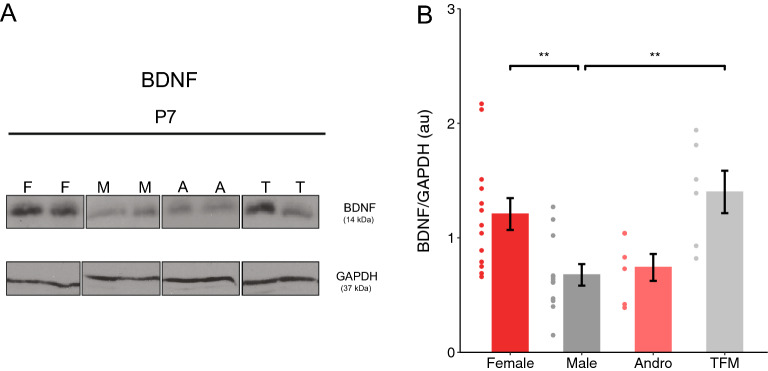
Perinatal testosterone negatively correlates with BDNF expression levels during the first postnatal week. (**A**) Western blot analysis of mature BDNF (14 kDa) expression levels in the hippocampus of different experimental groups at P7. Each lane represents a different animal. Blot shows representative samples of different sex groups (images were cropped from different gels). (**B**) Quantification revealed that the expression of mature BDNF is significantly lower in male when compared to female and testosterone-insensitive male rat pups (F: 1.21 ± 0.14 au, n = 13 rats from 4 litters; M: 0.68 ± 0.09 au, n = 10 rats from 4 litters; A: 0.74 ± 0.12 au, n = 6 rats from 3 litters; TFM: 1.4 ± 0.19 au, n = 6 rats from 3 litters; One-way ANOVA with Tukey’s post hoc analysis, F × M: p = 0.009; TFM × M: p = 0.007). Dots represent individual data points. *F* females, *M* males, *A* andro/testosterone-treated females, *T* TFM/testosterone-insensitive males. Graphs represent mean ± SEM.

Several studies have suggested that BDNF may play a role in the developmental increase of KCC2 expression^35–37^. Sex steroid hormones may induce BDNF transcription, enhance CREB activity and modulate it epigenetically^38–40^, which may account for the functional discrepancies in BDNF between different sexes. Since we found that KCC2 localization in the membrane of hippocampal neurons was hormone dependent, we asked whether BDNF expression levels in the hippocampus at P7 were hormone dependent as well. We found higher levels of BDNF in the hippocampus of females and testosterone-insensitive males compared to male rat pups (Fig. 5A, Supplementary Fig. S2B; One-way ANOVA with Tukey’s post hoc analysis, F × M: p = 0.009; TFM × M: p = 0.007). Thus, these data show that during the first postnatal week higher testosterone signaling was associated with lower BDNF expression levels.

### Perinatal testosterone affects spontaneous GABAergic neurotransmission in the adult hippocampus

**Figure 6:**
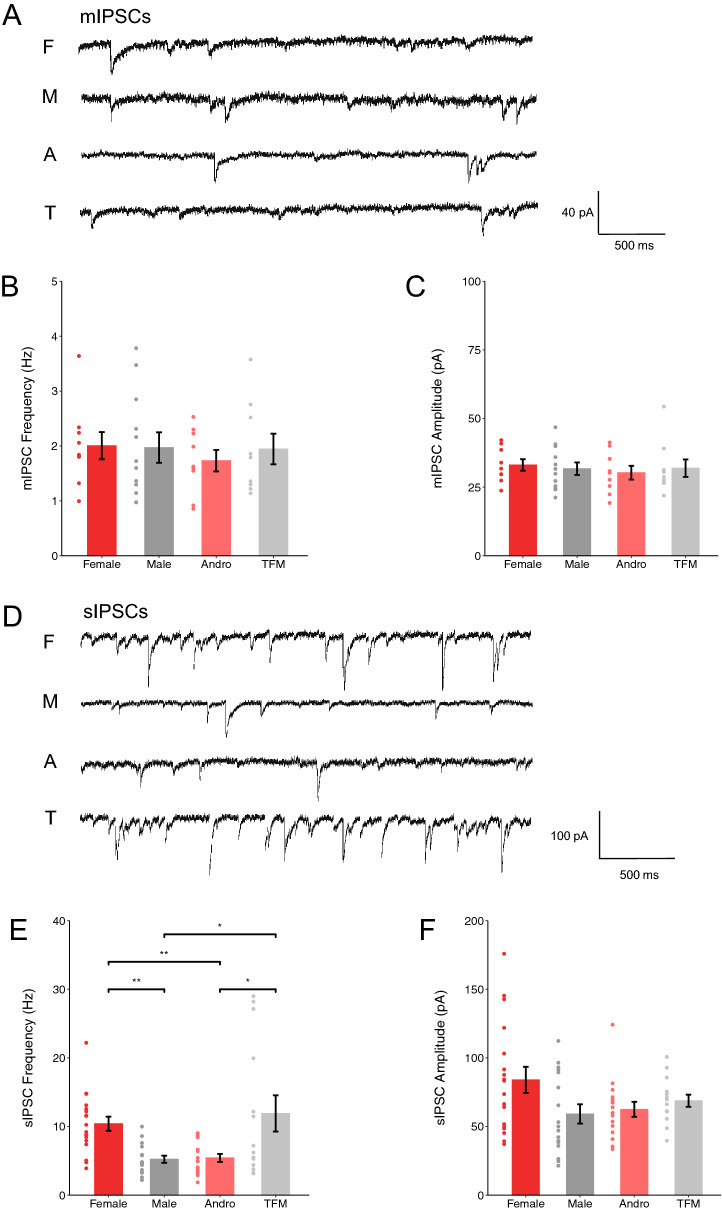
Testosterone signaling is associated with lower sIPSC frequency in young adult CA1 pyramidal neurons. (**A**) Representative traces of mIPSCs from CA1 pyramidal neurons. (**B**) mIPSC frequency (F: 2.01 ± 0.25 Hz, n = 9 cells, 4 rats from 3 litters; M: 1.97 ± 0.28 Hz, n = 12 cells, 5 rats from 3 litters; A: 1.73 ± 0.20 Hz, n = 9 cells, 4 rats from 3 litters; TFM: 1.95 ± 0.28 Hz, n = 9 cells , 4 rats from 3 litters; One-way ANOVA with Tukey’s post hoc analysis, p = 0.985) and (**C**) amplitude (F: 33.10 ± 2.10 pA, n = 9 cells, 4 rats from 3 litters; M: 31.70 ± 2.27 pA, n = 12 cells, 5 rats from 3 litters; A: 30.28 ± 2.52 pA, n = 9 cells, 4 rats from 3 litters; TFM: 31.92 ± 3.20 pA, n = 9 cells,4 rats from 3 litters; One-way ANOVA with Tukey’s post hoc analysis, p = 0.849) did not differ in hippocampal CA1 pyramidal neurons between the sex groups. (**D**) Representative traces of sIPSCs from CA1 pyramidal neurons. (**E**) Sex differences were found in the frequency of sIPSC (F: 10.40 ± 1.03 Hz, n = 18 cells, 7 rats from 4 litters; M: 5.23 ± 0.52 Hz, n = 17 cells, 6 rats from 3 litters; A: 5.42 ± 0.58 Hz, n = 16 cells, 6 rats from 3 litters; TFM: 11.90 ± 2.64 Hz, n = 14 cells, 5 rats from 3 litters; One-way ANOVA with Tukey’s post hoc analysis, F × M: p = 0.002; TFM × M: p = 0.021; F × A: p = 0.004; TFM × A: p = 0.030). (**F**) Conversely, no sex differences were found in the amplitude of sIPSC (F: 83.98 ± 9.48 pA, n = 18 cells, 7 rats from 4 litters; M: 59.13 ± 7.00 pA, n = 17 cells, 6 rats from 3 litters; A: 62.50 ± 5.45 pA, n = 16 cells, 6 rats from 3 litters; TFM: 68.74 ± 4.43, n = 14 cells, 5 rats from 3 litters; One-way ANOVA with Tukey’s post hoc analysis, p = 0.067) in the four experimental groups. Dots represent individual data points. *F* females, *M* males, *A* andro/testosterone-treated females, *TFM* testosterone-insensitive males. Graphs represent mean ± SEM.

To determine whether perinatal testosterone had long term consequences on GABAergic neurotransmission, we recorded miniature and spontaneous inhibitory postsynaptic currents (IPSCs) from pyramidal cells in the CA1 region of the dorsal hippocampus in P40 rats. We did not find any significant difference in mIPSC frequency (Fig. 6A,B; One-way ANOVA with Tukey’s post hoc analysis, p = 0.895) or amplitude (Fig. 6A,C; One-way ANOVA with Tukey’s post hoc analysis, p = 0.849) in the four experimental groups, suggesting that GABAergic synapse numbers or strength were not significantly affected by testosterone signaling during the perinatal period. Conversely, at the network level, we observed that the mean frequency of sIPSCs was significantly smaller in pyramidal neurons from males compared to pyramidal neurons from females and testosterone-insensitive males (Fig. 6D,E; One-way ANOVA with Tukey’s post hoc analysis, F × M: p = 0.002; TFM × M: p = 0.021). We further observed a decreased frequency of sIPSC in testosterone-treated females compared to females and testosterone-insensitive males (Fig. 6D,E; One-way ANOVA with Tukey’s post hoc analysis, F × A: p = 0.004; TFM × A: p = 0.030). sIPSC amplitude was not significantly different between the four experimental groups (Fig. 6D,F; One-way ANOVA with Tukey’s post hoc analysis, p = 0.067). Overall, these results suggest that perinatal testosterone signaling lead to overall reduced spontaneous inhibitory transmission in young adult rodents.

## Discussion

In the present report, we provide evidence that membrane KCC2 localisation is negatively regulated by perinatal testosterone signaling in the hippocampus during the first postnatal week. We also demonstrated that perinatal testosterone signaling leads to decreased spontaneous inhibitory transmission in adult hippocampus. These data urge caution in generalizing findings regarding the cellular and molecular mechanisms underlying GABAergic circuit development and function in the different sexes.

During early postnatal development, a shift from GABA_A_-mediated excitation to inhibition occurs in a wide array of brain structures, including the hippocampus^41,42^. The developmental GABA switch in the hippocampus has traditionally been studied only in male rats. In recent years, research using female rats has revealed precocious GABA_A_ transmission compared to male rats. In female rats, the GABA switch in CA1 and CA3 pyramidal neurons occurs between P4 and P7. In male rats, the transition occurs between P7 and P14^22,25,43–49^, suggesting a longer window of GABA_A_-mediated excitation in males compared to females. The mechanisms underlying these sex differences are poorly understood. It has been suggested that the testosterone surge that occurs perinatally in males^50^ promotes the expression and the activity of NKCC1, while decreasing the synthesis and activity of KCC2^51^, which would result in males experiencing the GABA shift later than females. This effect has been shown not only in hippocampus, but also in substantia nigra reticulata neurons in acute slices^52^, and embryonic hypothalamic neurons in culture^53^. Our data show that testosterone signaling does not seem to alter KCC2 mRNA or protein levels, whereas it significantly limits KCC2 localisation at the membrane in males and masculinized females. Membrane KCC2 levels correlate with the development of inhibitory neurotransmission^54^; on the other hand, cytoplasmic KCC2 does not seem to affect the reversal potential, E_GABA_^55^. Therefore, our results suggest that KCC2 transporter activity may play a larger role in influencing E_GABA_ in the female than in the male hippocampus during the first postnatal week. Functional evaluation of E_GABA_ in the different experimental groups would further support this hypothesis. Altogether, the changes we observed in KCC2 localisation show an organizational effect of gonadal steroids. These effects might be brain region specific, since for example the GABA shift in Purkinje cells in the cerebellum is delayed in females compared to males^56^.

One of the most powerful modulators of KCC2 activity is BDNF^37,57,58^. Here, we report that overall BDNF expression levels are significantly higher in females compared to males during the first postnatal week. Consistent with our data, quantification of BDNF concentrations showed higher levels of BDNF in the hippocampus, ventromedial hypothalamus, and cortex in female compared to male rats^59–62^. Lower BDNF levels may play a role in reduced KCC2 plasmalemmal localization in males because BDNF has been found to enhance KCC2 membrane confinement^35,58,63^. Further experiments are needed to determine whether different BDNF levels underlie the effects of perinatal testosterone signaling on KCC2 membrane localisation. Nevertheless, these data indicate that when investigating the effects of BDNF in vivo, the sex of the animal models employed is a crucial variable to consider.

Gonadal hormones have been shown to influence synaptic transmission via genetic processes as well as fast changes in cell-to-cell communication^64–68^. In gonadotropin-releasing hormone neurons, estrogens control GABA release and cause bursts in GABA_A_R-dependent inhibitory postsynaptic currents^69^. Through its impact on GABA_A_R, another ovarian hormone, progesterone, and its metabolite allopregnanolone, also regulate inhibitory neurotransmission^70,71^. Here, we report that CA1 pyramidal neurons from females and testosterone-insensitive males show higher sIPSC frequency at the end of adolescence, which indicates an increase of spontaneous basal inhibition. This could be due to increased GABAergic circuit excitability or/and increased drive of GABAergic circuits synapsing onto CA1 pyramidal cells. All together, these data suggest that organizational perinatal testosterone leads to long lasting effects on spontaneous GABAergic activity in post-adolescent brain. Higher sIPSC frequency may lead to an overall decrease, or enhanced stability, of neuronal excitability, which could in turn contribute to a more resilient state of female compared to male brains against pathological states, such as epilepsy^72,73^. Further studies will be critical to understand how gonadal hormones impact different aspects of neuronal development, thus contributing to sex bias in specific developmental and pathological conditions.

## Methods

### Experimental models and prenatal procedures

Sprague–Dawley wild-type (wt) rats and Sprague–Dawley rats carrying the *Tfm* allele were bred with commercially purchased Sprague–Dawley (Charles River, St. Constant, QC, Canada) and were group housed in our colony at Sainte-Justine Hospital Research Centre within a 12-h light/dark cycle with free ad libitum access to food and water. Animal care, use and procedures were conducted in accordance with the Canadian Council on Animal Care regulations and conformed to the guidelines of protocols #617 and #696, which were approved by Comité Institutionnel de Bonnes Pratiques Animales en Recherche (CIBPAR) at Sainte-Justine Hospital Research Centre and Université de Montréal (Montreal, Quebec, Canada). This study also complies with the ARRIVE guidelines.

The testicular feminization mutation (Tfm) carriers were provided by Dr. Cindy Jordan (Michigan State University, MI, USA). Tfm is a naturally occurring point mutation of the gene that codes for the androgen receptor (AR), rendering the Tfm male rat insensitive to physiological levels of androgens^30^. Genotype was determined by extracting DNA from ear punches followed by PCR to detect Tfm versus wild type (WT) alleles for androgen receptor (AR), and the presence or absence of the Sry gene, which is located on the Y chromosome^74^ (Fig. 2B). For this study, we used WT males, WT females that were not carrying the Tfm allele, and Tfm males. The primer sequences for genotyping are listed in Table 1.

**Table 1:** Sequence of primers used in the RT-qPCR and genotyping.

Target genes: RT-qPCR	Primers
α1	**F:** 5′-CGATCCTCTCTCCCACACTT-3′ **R:** 5′-TCTTCATCACGGGCTTGTC-3′
α5	**F:** 5′-CACCCAACAAGCTGCTGA-3′ **R:** 5′-GACACTCAGCAGAGATCGTCA-3′
β2	**F:** 5′-CTGGGTCTCCTTTTGGATCA-3′ **R:** 5′-TCATCGTCAGGACAGTTGTAATTC-3′
γ2	**F:** 5′-TGCTCACTGGATCCGACTC-3′ **R:** 5′-GTAATTGCAACTGGCACTCG-3′
GAD65	**F:** 5′-AGGCTCTGGCGATGGAAT-3′ **R:** 5′-TTATAGCGGGCAATGAGCA-3′
GAD67	**F:** 5′-GCGAGAGATCATTGGATGGT-3′ **R:** 5′-GCCATGATGCTGTACATGTTG-3′
GAT-1	**F:** 5′-GATTGGCCTCTCTAACATCACC-3′ **R:** 5′-CAAAGAAAACCAGGAACAGCA-3′
KCC2	**F:** 5′-GGAGTCCTTCTGCATGGTCT-3′ **R:** 5′-ATGGAAATGGCTGTGAGCAT-3′
NKCC1	**F:** 5′-CAGCAATAGCTACCAATGGATTC-3′ **R:** 5′-TGGCCCTAGACTTCTAGAGATTAAA-3′

Reference genes	Primers
Actb	**F:** 5′-CCCGCGAGTACAACCTTCT-3′ **R:** 5′-CGTCATCCATGGCGAACT-3′
Gapdh	**F:** 5′-CCCTCAAGATTGTCAGCAATG-3′ **R:** 5′-AGTTGTCATGGATGACCTTGG-3′
Hprt	**F:** 5′-GACCGGTTCTGTCATGTCG-3′ **R:** 5′-ACCTGGTTCATCATCACTAATCAC-3′

Target genes: genotype	Primers
AR	**F:** 5′-GGCTGTGTGAGGGCCAAATT-3′ **R:** 5′-GGACCAAAGGCTGATCACAAG-3′
SRY	**F:** 5′-GGGAGGAGGGATGAATAT-3′ **R:** 5′-CATTGCAGCAGGTTGTACAGT-3′

Pregnant dam body weight was measured from gestational day (GD) 1 (day of plug) to 14 to explore the rate of daily body weight gain during pregnancy. At GD14, dams were randomly assigned to one of the following treatments:

- Vehicle: dams were injected s.c. with vehicle solution (sesame oil: 0.1 ml/rat) from GD14 to 19.
- Testosterone: dams were injected s.c. with a 1 mg/0.1 ml of testosterone propionate solution (Testosterone propionate, MP Biomedicals, cat: 218655, lot: 40272, dissolved in Sesame oil) from GD14 to 19. Testosterone regimen was based on Wolf et al.^31^. All injections were performed at 1 PM during the light phase of the light/dark cycle. On the day of parturition, anogenital distance (AGD) was recorded. Rats were weaned at P21. Male and female offspring were housed in separate cages, with no more than five pups per cage, with standard rat chow and water ad libitum.

### Sexual developmental markers

Sexual developmental markers were measured as described in Pallarés et al.^75^. AGD was measured using a vernier-caliper at P1. On P15, pups were re-examined for sexual phenotype, their sex confirmed or reassigned if necessary, and males and females were checked for areolas in a blind fashion. Areolas were deemed as either faint or normal, i.e. prominent and easily identified. On P35, female offspring were checked for vaginal opening (VO) and male offspring were monitored for testicular descent as indicators of puberty. Tfm males were dissected at P40 to confirm the presence of testis internally.

### Hormone assays

Trunk blood was collected at birth, centrifuged to separate the plasma, and analyzed for testosterone and estradiol levels. Testosterone and estradiol concentrations were measured using AlphaLISA kit (Perkin Elmer, cat: AL324, lot: 2477098) and ELISA kit (Abcam, cat: ab108667, lot: GR3214106-3), respectively, following the company’s protocol.

### RT-qPCR

Total RNA was extracted using the “Aurum Total RNA fatty and fibrous tissue” kit and following the steps outlined in “Section 8: Spin protocol” of the instruction manual (732-6830, Bio-rad). The quality of extracted total RNA was assessed using a Bioanalyzer (CHUSJ, Montreal, QC, Canada) and conformed with high purity and integrity standards to perform RT-qPCR. The reverse transcription of RNA into cDNA and the following qPCR were performed in collaboration with IRIC (Institute for Research in Immunology and Cancer, Montreal, QC, Canada). Three endogenous controls (ACTB, HPRT, GAPDH) were tested and GAPDH being the more stably expressed across samples was chosen for the final analysis as the reference gene. Details of RT-qPCR primer sequences are listed in Table 1.

### Western blot

Whole lysate proteins and membrane protein fractions were extracted from hippocampal tissue at P7 and P40 following previously described protocols^58,76^. In particular, to obtain membrane protein fractions, samples were homogenized in 5 vol of HB (300 mM sucrose/10 mM Tris–HCl, pH 7.5/1 mM EDTA/protease inhibitor mixture) and centrifuged at 1000×*g* for 10 min at 4 °C. The pellet was washed in 0.5 vol HB and used as the nuclear fraction. Supernatants were centrifuged at 17,000×*g* for 15 min at 4 °C, yielding the mitochondria fraction. The supernatant was further separated by ultracentrifugation at 100,000×*g* for 1 h. The pellet and supernatant were used as the membrane and cytosol fractions, respectively. Membranes were probed with the following primary antibodies: anti-KCC2 1:1000 (rabbit polyclonal IgG; Cat. no. 07-432, Millipore), 1:200 anti-BDNF (BDNF, mouse monoclonal IgG, Cat. no. 327100, Icosagen), anti-glyceraldehyde-3-phosphate dehydrogenase 1:4000 (GAPDH, mouse monoclonal IgG; Cat. no. AM4300; Applied Biosystems) and anti-β dystroglycan 1:3000 (rabbit polyclonal IgG, Cat no. ab43125, Abcam). All samples were run simultaneously. Bands were quantified using ImageJ v.1.52 software (National Institutes of Health, USA, http://imagej.nih.gov/ij). The intensity of KCC2 and BDNF bands were normalized over the intensity of the GAPDH band for whole lysates or of the β dystroglycan band for membrane fractions, in the same lane (internal loading control). We did not quantify KCC2 dimer band because it was not detectable in all samples. Full gels of western blot with molecular weight markers are shown in Supplementary Figs. S1 and S2. During all experimental steps, the experimenter was blinded to the treatment groups.

### Hippocampal slice preparation and in vitro electrophysiology

Brain slices were prepared as previously described by Sanon et al.^77^. Briefly, WT rats aged 38–42 days old were anaesthetized with isoflurane (Baxter corp, Mississauga, ON, Canada) and rapidly decapitated. The brain was extracted and immersed in cold (4 °C) and oxygenated (95% O_2_, 5% CO_2_) high sucrose cutting artificial cerebro-spinal fluid (ACSF) containing (in mM): 250 sucrose, 2 KCl, 0.5 CaCl_2_, 26 NaHCO_3_, 1.25 NaH_2_PO_4_, 7 MgSO_4_·7H_2_O, 10 D-glucose. Transverse cortical slices (300 µm) were cut using a vibratome (VT1000S, Leica Microsystems Inc., Buffalo, NY, USA) and placed in oxygenated ACSF containing (in mM): 126 NaCl, 3 KCl, 2 CaCl_2_, 25 NaHCO_3_, 1 NaH_2_PO_4_, 2 MgSO_4_·7H_2_O, 10 D-glucose (pH 7.3–7.4; 300–310 mOsm) at room temperature. Slices with coordinates − 3.08 to − 4.36 mm posterior to bregma from both hemispheres were allowed to recover a minimum of one hour before individual slices were transferred in a submerged recording chamber with ACSF flowing at a rate of 2 mL/min.

Whole-cell recordings were made using 1 mm (outer diameter) borosilicate patch pipettes (A-M Systems, Carlsborg, WA, USA) yielding a series resistance of 4–7 MΩ when filled with an intracellular solution containing (in mM) 140 CsCl, 5 NaCl, 2 MgCl_2_, 10 HEPES, 0.5 EGTA, 10 phosphocreatine, 2 ATP-tris, 0.4 GTP-Li (pH 7.2–7.3 adjusted with CsOH, 290 mOsm). Signals were acquired using an Axopatch 200B amplifier (low-pass filtering at 1 kHz). Data acquisition, at a sampling rate of 5 kHz was performed using a Digidata 1440A analog–digital converter (Molecular Devices, Sunnyvale, CA, USA) and pClamp10 software (Molecular Devices, Sunnyvale, CA, USA). We recorded from visually identified CA1 pyramidal cells of the hippocampus using an upright microscope (BX50WI, Olympus Canada, Markham, ON, Canada) equipped with differential interference contrast and infrared (DIC-IR) CCD video camera (KP M1U, Hitachi Denshi Ltd, Japan). To investigate the inhibitory activity onto hippocampal pyramidal cells, we recorded spontaneous inhibitory postsynaptic currents (sIPSC) in voltage-clamp mode at a membrane potential of − 60 mV and in presence of 6,7-dinitroquinoxaline-2,3-dione (DNQX, 40 μM), 2-amino-5-phosphonopentanoic acid (D-AP5, 50 μM), and routinely blocked these currents with bicuculline (BIC, 2 μM). Miniature inhibitory post-synaptic currents (mIPSCs) were measured in the presence of 0.5 μM TTX. Intrinsic properties, such as resting membrane potential, input resistance and capacitance were also analyzed. During all electrophysiological recordings, the experimenter was blinded to the treatment groups.

### Statistical analysis

Statistical analysis and generation of figures was performed with RStudio (RStudio Inc., version 1.2.1335). Data was log-transformed to fit a normal distribution and assessed via one-way ANOVA for multiple comparisons between groups. Post-hoc Tukey’s test was performed to identify statistically significant differences when significant main effects were detected. The accepted threshold of significance for all statistical tests was set at a two-tailed *p*-value < 0.05. In particular, significance was considered as *p*-value < 0.05 and fold change > 2 or < 0.5 for all comparisons of qPCR data. All data are presented as mean ± standard error of the mean (SEM).

## Supplementary Information

Supplementary Figures.
