# Supplementary material for: Sex-specific differences in KCC2 localisation and inhibitory synaptic transmission in the rat hippocampus
Source: Sci Rep. 2022 Feb 24;12:3186. doi: 10.1038/s41598-022-06769-5 (PMC8873453; doi:10.1038/s41598-022-06769-5)
Supplement: Supplementary file 1 — Supplementary Figures. [file 41598_2022_6769_MOESM1_ESM.docx]

**Sex-specific differences in KCC2 localisation and inhibitory synaptic transmission in the rat hippocampus**

Daniele C. Wolf, Nathalie T. Sanon, Alexandra O. S. Cunha, Jia-Shu Chen, Tarek Shaker, Abdul-Rahman Elhassan, Antônia Sâmia Fernandes do Nascimento, Graziella Di Cristo, Alexander G. Weil

**Supplementary Figure 1**

**
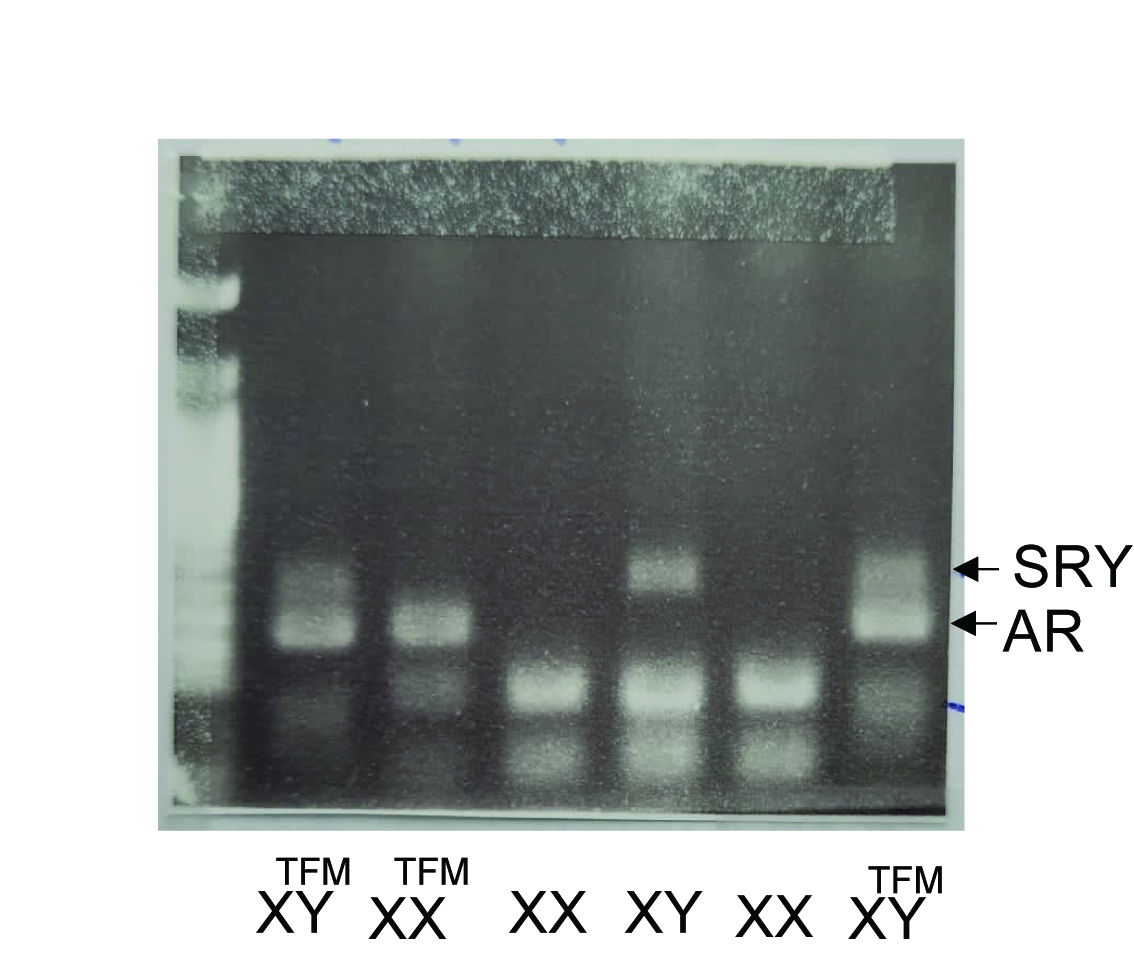
**

**Figure S1**. Original full-length image of polyacrylamide gel with the amplification and digestions results of the AR and SRY genes in WT females, WT males, Tfm males and Tfm female carriers showed in Figure 2B of the manuscript.

**Supplementary Figure 2
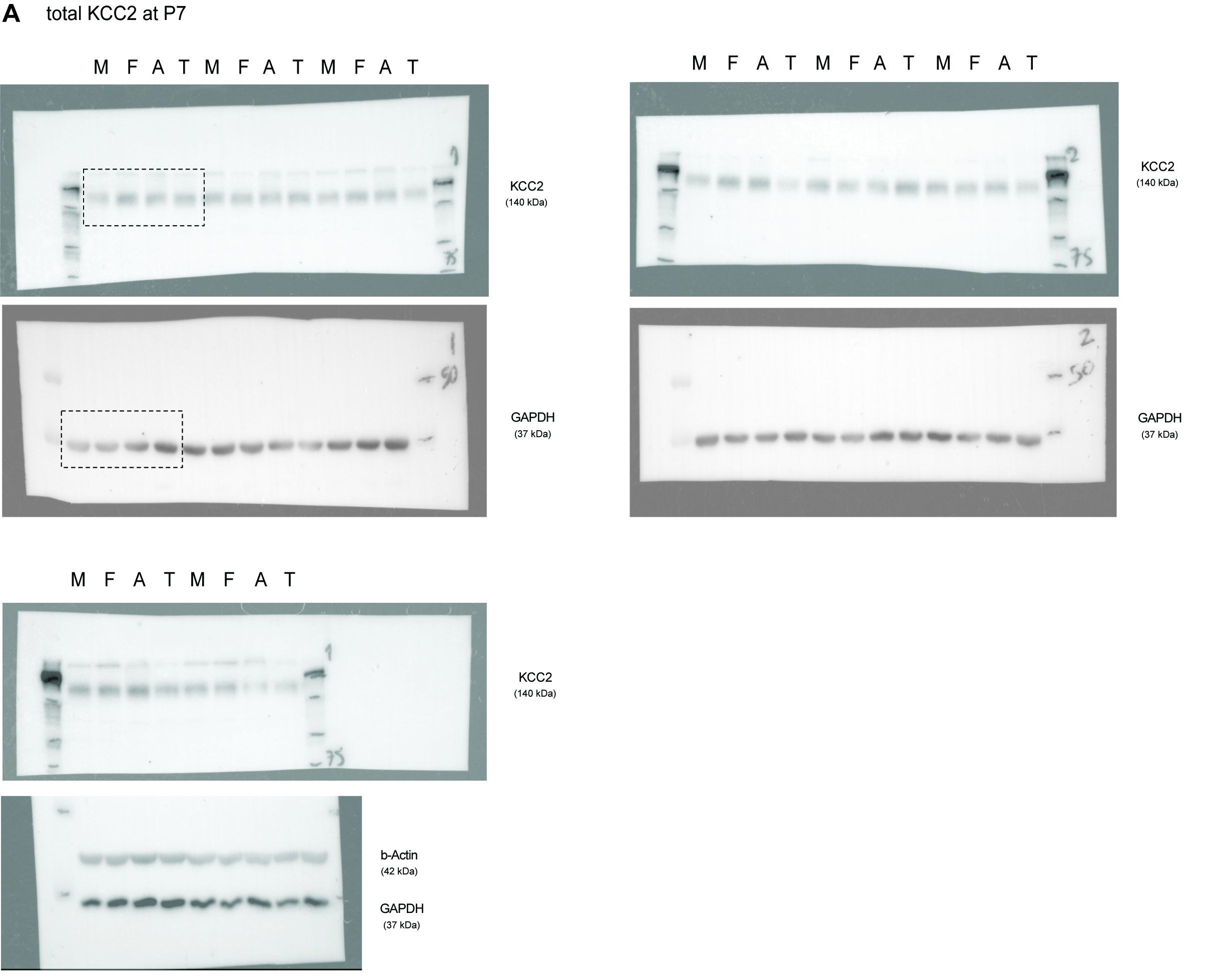
**

**
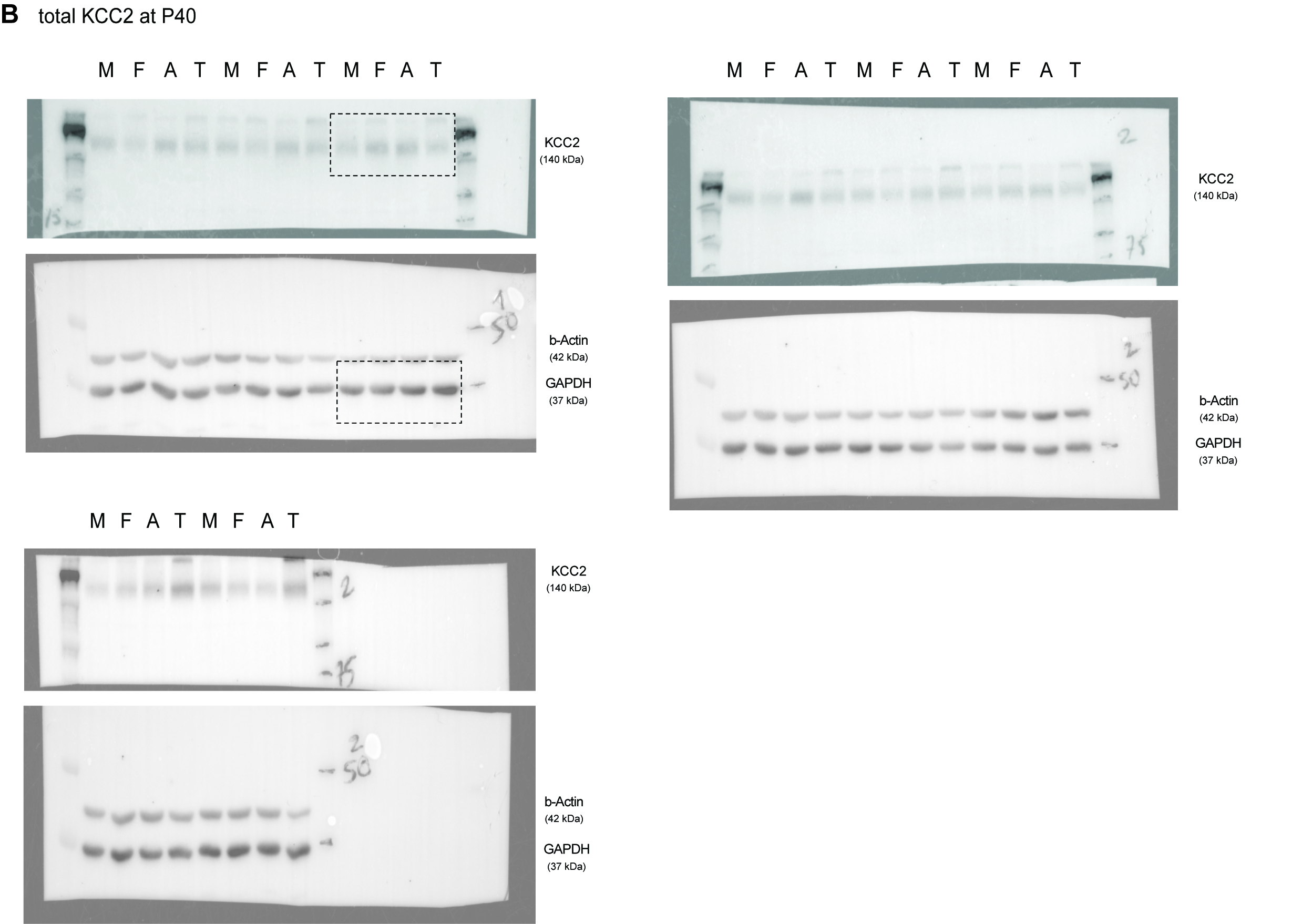
**

**Figure S2. A, B.** Uncropped full-length pictures of western blot membranes used for the quantification of total KCC2 expression at P7 (A) and P40 (B). Dotted areas indicate lanes shown in Figure 4A. Blots were developed by chemiluminescence. Membranes were cut to enable blotting for different antibodies. Note that we did not quantify KCC2 dimer band because it was not reliably detectable in all samples. F: females; M: males; A: andro/testosterone-treated females; T: TFM/testosterone-insensitive males.

**Supplementary Figure 3**

**
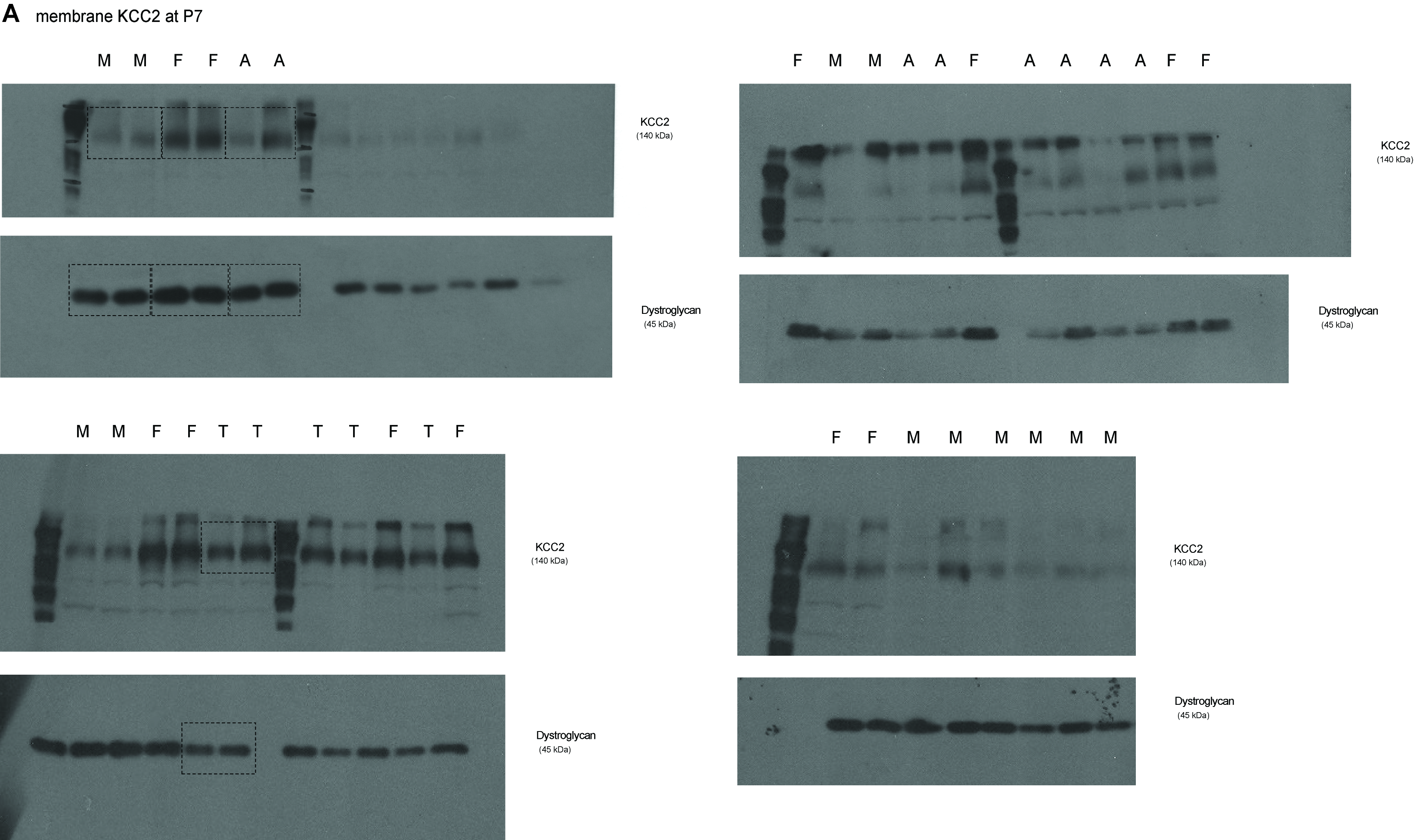
**

**
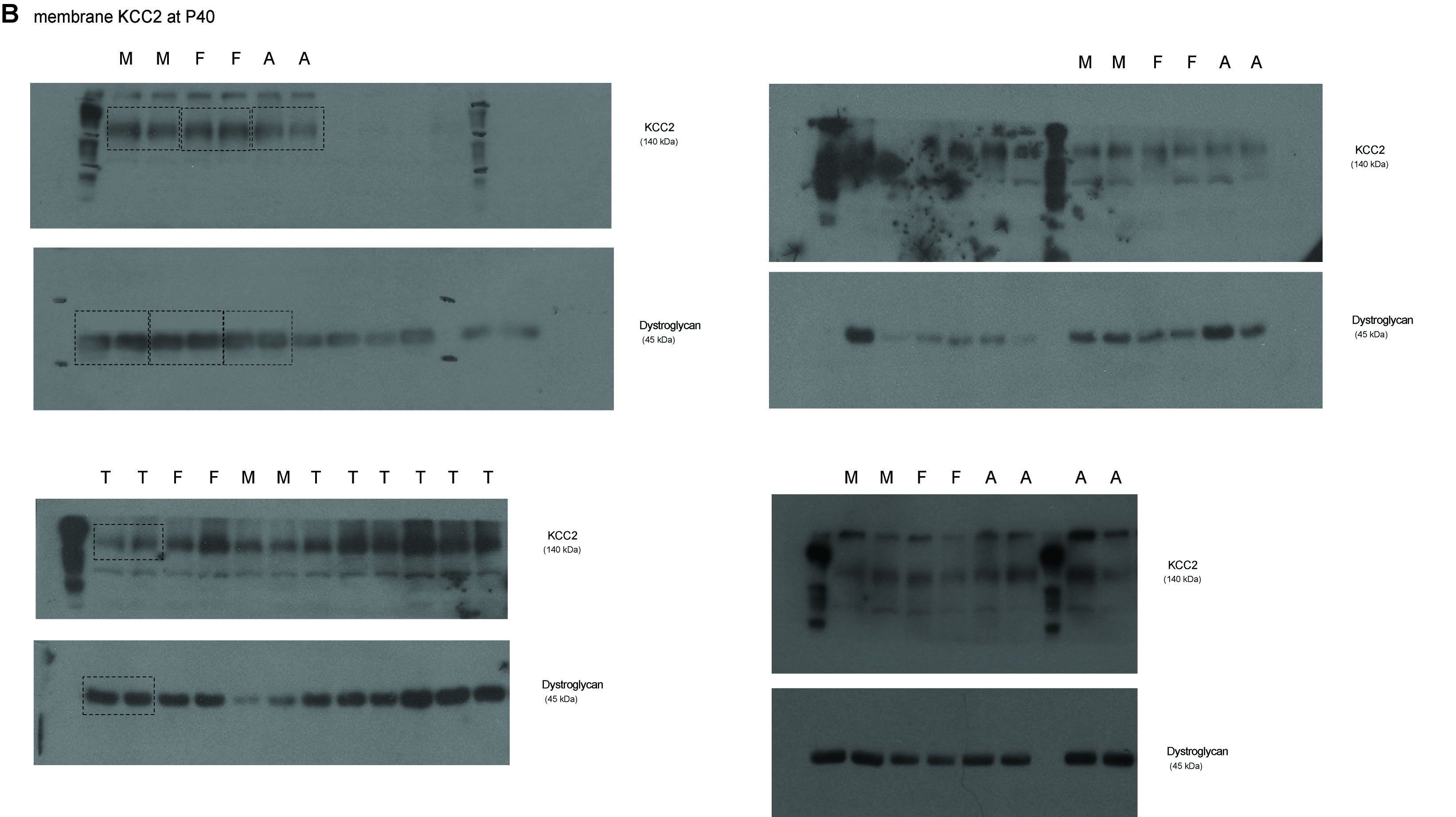
**

**Figure S3. A, B.** Original full-length pictures of western blot membranes used for the quantification of membrane KCC2 expression at P7 (A) and P40 (B). Dotted areas indicate lanes shown in Figure 4D. Blots were developed using X-rays films. Membranes were cut to enable blotting for different antibodies. Note that we did not quantify KCC2 dimer band because it was not reliably detectable in all samples. F: females; M: males; A: andro/testosterone-treated females; T: TFM/testosterone-insensitive males.

**Supplementary Figure 4**


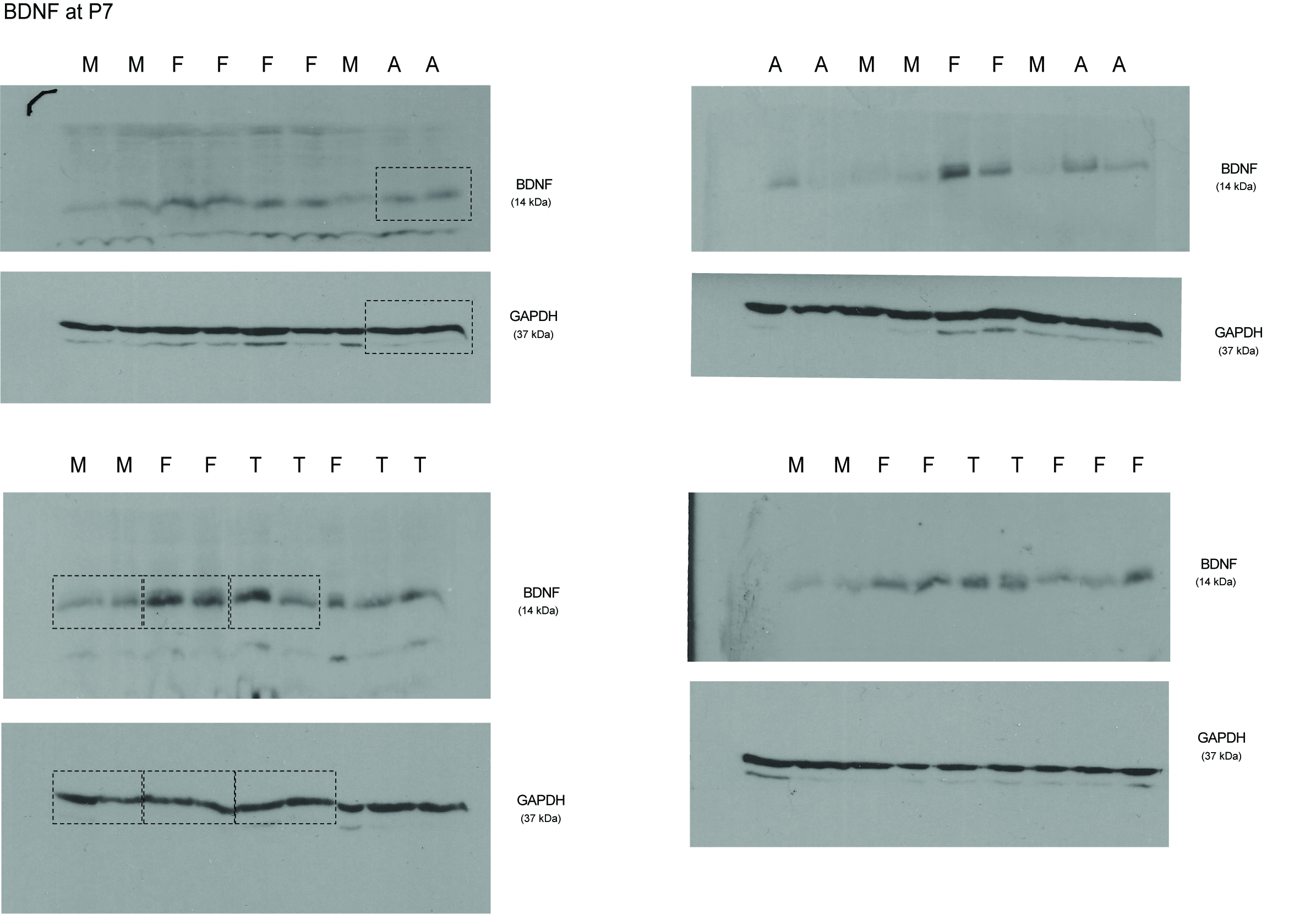


**Figure S4**. Original full-length pictures of western blot membranes of BDNF expression at P7. Dotted areas indicate lanes shown in Figure 5A. Blots were developed by X-rays films. Membranes were cut to enable blotting for different antibodies. F: females; M: males; A: andro/testosterone-treated females; T: TFM/testosterone-insensitive males.
